# High pregnancy incidence and low contraceptive use among a prospective cohort of female entertainment and sex workers in Phnom Penh, Cambodia

**DOI:** 10.1186/s12884-018-1768-3

**Published:** 2018-05-03

**Authors:** Putu Duff, Jennifer L. Evans, Ellen S. Stein, Kimberly Page, Lisa Maher, on behalf of the Young Women’s Health Study Collaborative, John Kaldor, Serey Phal Kien, Lisa Maher, Joel Palefsky, Kimberly Page, Vonthanak Sapphon, Mean Chhi Vun

**Affiliations:** 1 British Columbia Centre for Excellence in HIV/AIDS, St. Paul’s Hospital, 608-1081 Burrard Street, Vancouver, BC Canada; 2 Department of Medicine, University of British Columbia, St. Paul’s Hospital, 608-1081 Burrard Street, Vancouver, BC Canada; 3 Institute for Global Health, Department of Epidemiology, University of California-San Francisco, San Francisco, California USA; 4 Division of Epidemiology, Biostatistics and Preventive Medicine, Department of Internal Medicine, University of New Mexico Health Sciences Center, Albuquerque, NM USA; 5 Kirby Institute for Infection and Immunity (formerly the National Centre in HIV Epidemiology and Clinical Research), UNSW Australia I, Wallace Wurth Building, Sydney, NSW 2052 Australia; 6 Cambodian Women’s Development Association (CWDA, No. 19, Street 242, Boeung Prolit, Khan 7 Makara, Phnom Penh, Cambodia; 7 Department of Medicine, University of California San Francisco, 513 Parnassus Ave; UCSF, San Francisco, CA USA; 8 National Center for HIV/AIDS, Dermatology and STDs (NCHADS), #245H, Street 6A, Phum Kean Khlang, Sangkat Prekleap Russey Keo, Phnom Penh, Cambodia

**Keywords:** Pregnancy, Reproductive health, Sex work, Cambodia, Cohort study, prevention trials

## Abstract

**Background:**

While HIV and unintended pregnancies are both occupational risks faced by female sex workers, the epidemiology of pregnancy and its drivers in this population remains understudied. This includes Cambodia, where the drivers of pregnancy among female entertainment and sex workers (FESW) remain unknown. The current study aimed to examine factors associated with incident pregnancy, as well as describe contraceptive use among FESW in Phnom Penh, Cambodia.

**Methods:**

This analysis drew from the Young Women’s Health Study (YWHS)-2, a 12-month observational cohort of 220 FESW aged 15–29 years, conducted between August 2009 and August 2010. Interviewer-administered questionnaires were conducted at baseline and quarterly thereafter, alongside HIV and pregnancy testing. Bivariate and multivariable extended Cox regression analysis was used to examine correlates of incident pregnancy.

**Results:**

At baseline, 6.8% of participants were pregnant, and only 10.8% reported using hormonal contraceptives, with 11.3% reporting an abortion in the past 3 months. Pregnancy incidence was high, at 22/100 person-years (95% CI: 16.3–30.1). In multivariable analysis, younger age (19–24 years versus 25–29 years) (Adjusted Hazards Ratio (AHR): 2.28; 95% Confidence Interval (CI) 1.22–4.27), lower income (400,000–600,000 Riel (≤150$USD) versus > 600,000 Riel (> 150$USD)) (AHR 2.63; 95% CI 1.02–6.77) positively predicted pregnancy, while higher self-reported condom self-efficacy were associated with reduced pregnancy incidence (AHR 0.89; 95% CI 0.81–0.98).

**Conclusions:**

Results document high incidence of pregnancy and unmet reproductive health needs among FESWs in Cambodia. Findings point to an urgent need for multi-level interventions, including venue-based HIV/STI and violence prevention interventions, in the context of legal and policy reform. High pregnancy incidence in this population may also undermine recruitment and retention into HIV prevention intervention trials. The exploration of innovative and comprehensive sex worker-tailored sexual and reproductive health service models, also as part of HIV prevention intervention trials, is warranted.

## Background

Pregnancy in women at high risk of HIV is an important issue, with unprotected sex as the primary exposure for both HIV and pregnancy. While previous epidemiological research among women engaged in transactional sex has largely focused on risk of HIV and other sexually transmitted infections (STIs), a growing number of studies have begun to examine the broader reproductive health need of women engaged in transactional sex, including pregnancy [1–4]. Recent studies among women in engaged in transactional sex have documented lifetime pregnancy prevalence ranging between 70% and 90% [1, 3, 5–7], many of which are unintended pregnancies [7, 8]. The prevalence of unintended pregnancies among women engaged in transactional sex is high - up to 86% in some studies [3] –yet recent research indicates that many women engaged in transactional sex have a strong desire to have children [9], with pregnancy intentions similar to those of women in other occupations [10].

The high prevalence of pregnancy among women engaged in transactional sex also has important implications for the success of HIV prevention trials, which often enroll high-risk sexually active women, some of which target women engaged in transactional sex. Understanding the incidence and correlates of pregnancy among women involved in transactional sex is therefore critical, as pregnancy intention is often an exclusion criterion and actual pregnancy typically results in study product discontinuation (if the study product is considered potentially harmful to a pregnant woman or her fetus), potentially impacting the success of the trial, as well as the efficacy of subsequent interventions. Increased understanding of the drivers of pregnancy among women engaged in transactional sex is critical in developing sexual and reproductive health (SRH) services tailored to the specific needs of women engaged in transactional sex [11]. However, there remains a research gap on the prevalence and correlates of pregnancy among women working in entertainment venues with high-risk sexual patterns as well as women engaged in commercial sex. The few studies that have examined correlates of pregnancy, have found that individual and interpersonal factors such as previous history of abortion, having a steady intimate partner [12], experiencing violence [13] and alcohol/illicit drug use [12, 14] were associated with unintended pregnancy in this population.

In Cambodia, women engaged in high-risk and transactional sex are a heterogeneous group working in variety of environments and venues, whom collectively can be referred to as female entertainment and sex workers (FESW) [15, 16]. Empirical data on pregnancy among FESW remain limited. Previous work by Delvaux [8] and colleagues highlighted low knowledge around SRH and limited access to reproductive health services among brothel-based women engaged in transactional sex in Phnom Penh, underscoring a need to examine the drivers of pregnancy in this population. Moreover, little is known about the pregnancy outcomes of FESW following the implementation of the 2008 ‘Law on Suppression of Human Trafficking and Sexual Exploitation’ [17]. The ‘Trafficking Law’ effectively criminalized the selling and purchase of sex, by prohibiting almost all aspects of sex work including: public solicitation, procurement of prostitution, management of a sex work venue and the provision of premises for prostitution [18]. The passage of this law resulted in increased police crackdowns and brothel closures, and led to the displacement of FESW to entertainment venues, streets and guesthouses, where they lacked protections from peers and management [16]. In these new settings, FESW have reported increased vulnerability to client-perpetrated violence and a reduced ability to negotiate client condom use [16]. The displacement of FESW to diverse settings and the associated disruption of peer networks has been found to reduce the reach of HIV prevention and service delivery [16, 19], access to health services and condoms [16], and may also have had an impact on unintended pregnancy rates.

Given the dearth of research examining the correlates of pregnancy, particularly within the Cambodian context, this longitudinal study aimed to determine the factors associated with time to pregnancy among FESWs working in Phnom Penh, Cambodia over a twelve-month period. While our overall goal was to determine the set of factors that best describe incident pregnancy, we hypothesized that the following could potentially be associated with reduced time to pregnancy: marital status, young age and low parity, alcohol/illicit drug use, intimate partner violence and condom non-use by non-paying partners and clients.

## Methods

### Study design and setting

The data from this study were drawn from the Young Women’s Health Study 2 (YWHS-2), a prospective cohort of young women engaged in sex work across a variety of venues and settings in Phnom Penh, Cambodia. [15] The YWHS was led by a multidisciplinary team including Cambodian Women’s Development Association (CWDA), the National Center for HIV/AIDS, Dermatology and STD (NCHADS), the University of California, San Francisco (UCSF) in the United States, and the Kirby Institute at the University of New South Wales (UNSW) in Australia.

The study design and recruitment procedures are described in detail elsewhere [15]. Briefly, eligibility criteria included: being biologically female (aged 15–29), Khmer language comprehension, working in a sex work or entertainment venue and having two or more sexual partners in the last month *or* engaging in transactional sex (defined as having exchanged sex for money, drugs, or other goods or services) in the past three months, with plans of remaining in Phnom Penh throughout the course of the one-year study. Trained staff members recruited a convenience sample of women engaged in sex work from a variety of locations including: 1) YWHS information meetings held by CWDA; 2) neighbourhood-based outreach; and 3) referrals by previous participants or community groups. All study participants provided voluntary informed consent prior to enrolment in the study.

### Data collection

Between 2009 and 2010, study participants visited the YWHS clinic at baseline and quarterly thereafter, where they completed a structured questionnaire, administered in Khmer by trained interviewers. The questionnaire elicited a wide range of information including socio-demographic characteristics (e.g., age, education, marital status), individual and interpersonal factors such as alcohol and drug use, interpersonal violence as well as sex work factors including occupational and sexual risk history, reproductive health and STI/HIV prevention (e.g., STI infection, contraceptive use and pregnancy). Staffed by a team of health care professionals, including a physician, nurses, counselors and a laboratory technician, all study participants attending the YWHS clinic for interviews were also tested for HPV and provided with STI treatment free of charge. Participants who tested positive for HPV or HIV were referred to local health providers for no-cost medical assessment and treatment.

All participants underwent voluntary client-centred risk-reduction counseling prior to testing. HIV serology testing, using two rapid tests: Uni-Gold Recombigen HIV rapid HIV test (Trinity Biotech USA, Jamestown, New York, USA) and the Clairview HIV ½ STAT-PAK (Inverness Medical Diagnostics, Waltham, Massachusetts, USA). HIV-1 immunoblot was used to confirm HIV serodiscordant samples. All participants who tested negative for HIV and were not pregnant were offered HPV vaccination. Study participants were provided free transport to interview sites and remunerated (US$5) for their time and expertise, regardless of whether they had or had not received the HPV vaccine. Ethical approval was obtained from the Institutional Review Board of the Committee on Human Research at the University of California, San Francisco, the Cambodian National Ethics Committee and the University of New South Wales Human Research Ethics Committee, aligned with both national and institutional ethical standards and the Helsinki Declaration of 1975 (revised in 2000). The Cambodian and U.S. IRB deemed the inclusion of minors ethical and waived requirement for parental consent based on the minimal risk posed by study procedures and potential for direct benefit to participants. Informed consent procedures following international guidelines (Declaration of Helsinki, and Council for International Organizations of Medical Sciences (CIOMS) with WHO)) were conducted in Khmer, by trained research personnel and with ongoing consultative supervision and training from staff of Cambodia National Center for HIV AIDS, and STDs (NCHADS) STD Clinic in Phnom Penh, Cambodia.

### Measures

#### Dependent variable

The primary dependent variable, incident pregnancy, was based on a ‘Yes’ response to the question, ‘As far as you know, are you pregnant now?’ which was asked at each three-monthly follow-up.

#### Independent variables

Independent variables were considered based on a priori knowledge of their associations with pregnancy, including among women engaged in transactional sex, in the literature. Age and education (number of years in school) were considered fixed variables, and all other variables were considered time-variant and updated at every quarterly follow-up. Other socio-demographic variables included marital status (defined as living together (married), living together (not married) and living alone (divorced, widowed and single)) and number of children (measured continuously). Individual sexual and substance use patterns were also considered, such as number of days participants drank alcohol in the last month (measured continuously), self reported amphetamine-type stimulant (ATS) use in the last 3 months, including crystal (ice) and *yama*. We also included a 6-item scale for measuring self-efficacy for negotiating condom use, modified for the sex work context [20]. The scale included questions such as: “I can ask a new private partner to use condoms” and “I can refuse sex when I don’t have a condom available”. Participants indicated their agreement with each of the five items measured on a five-point Likert scale ranging from strongly agree (for a score of 4) to strongly disagree (for a score of 0). A continuous score for condom self-efficacy was used, by summing the scores for all five items. Sex work variables included: number of sex clients in the past month and income in the last month. Women indicated if they currently worked as: a beer promoter, in a beer garden, as a waitress or hostess in a karaoke bar, nightclub, in a massage parlour, brothel, or as a freelance worker in the park, street or another location. FESWs were categorized as entertainment venue-based (i.e., working as beer promoter, in a beer garden, as a waitress or hostess in a karaoke bar, nightclub, in a massage parlour) versus non-entertainment venue-based (i.e., working on the street, brothel or public spaces). In light of the literature linking gender-based violence (from clients and intimate partners) with reduced condom negotiation, use and pregnancy [21, 22], we also examined the relationship between physical and sexual violence by clients and intimate partners, in the last year.

### Statistical analyses

Pregnancy incidence was calculated using the person-years method. Frequencies and proportions were calculated at baseline for categorical variables, and measures of central tendency (i.e., mean, median and IQR) were used to describe continuous variables. To examine the relationships between potential confounders on time to pregnancy, time-dependent Cox regression analyses were used. First, bivariate analyses using an extended Cox regression model were run to estimate the time to pregnancy using unadjusted hazard ratios. Using a conservative *p*-value cut-off of 0.10, a priori potential confounders that were also significant at *p* < 0.10 were included in a full multivariable model (model 1). A second model was constructed with variables significant at *p* < 0.05 in bivariate analysis (model 2). All statistical analyses were performed using SAS software version 9.3 (SAS Institute, Cary, NC, USA).

## Results

A total of 204 FESWs were included in our sample, yielding 799 observations over the one-year/12-month follow-up period. More than two thirds of participants (60.3%) were between the ages of 25–29, with 119 (58.3%) reporting between one to six years of education. Most participants (63.2%) were single, divorced, widowed or separated and the median number of children was three (IQR: 2–4) (*See* Table 1). At baseline, 14 (6.8%) participants were pregnant, and only 22 (10.8%) reported using hormonal contraceptives (i.e., oral contraceptive pills or injectable hormones), and 23 (11.3%) reporting an abortion in the past 3 months *(*Table 2*)*. Pregnancy incidence was high, at 22/100 person-years (py) (95% CI: 16.3–30.1). The number of new pregnancies at each quarterly time point were as follows: 13, 9, 6 and 13. In bivariate analysis, variables associated with increased pregnancy incidence (at *p* < 0.10) included: age (19–24 years versus 25–29 years); income in the last month (≤600,000 Riel versus > 600,000 Riel; the equivalent of ≤ $USD 150 versus >&USD 150); numbers of years in school (1–6 years versus 7+ years), cohabiting with a partner (not legally married) compared to living alone (single, widowed, divorced or separated) and hormonal contraceptive use in the last 3 months (measured at baseline). Factors associated with lower pregnancy incidence were HIV-positive serostatus, condom self-efficacy and condom use at last sex.

**Table 1:** Baseline characteristics of 204 Female and Entertainment Sex Workers (FESWs) in the YWHS-II study, Phnom Penh, Cambodia

Characteristic	Total (%) (*n* = 204)	Participants who were pregnant at least once over follow-up Yes (%) (*n* = 41)	*p*-value
Socio-demographics, biological and individual factors			
Age (years)			
16–18	14 (6.9)	2 (14.3)	0.997
19–24	67 (32.8)	20 (29.9)	0.010
25–29	123 (60.3)	19 (15.5)	–
Income, last month ($USD)			
≤ 150	128 (63.1)	26 (20.3)	0.061
> 150	75 (36.9)	15 (20.0)	–
Education			
No schooling	44 (21.6)	7 (15.9)	0.610
1–6 years	119 (58.3)	30 (25.2)	0.096
7+ years	41 (20.1)	4 (9.8)	–
Marital status			
Legally married	8 (3.9)	1 (12.5)	0.321
Living together, not legally married	67 (32.8)	17 (25.4)	0.026
Single, widowed, divorced or separated	129 (63.2)	23 (17.8)	–
Number of previous children (continuous) *Median, IQR*	3 (2–4)	3 (2–3)	0.079
HIV status			
HIV positive	33 (16.2)	3 (7.3)	0.086
HIV negative	171 (83.8)	38 (92.7)	–
Days drank alcohol, last month	15 (3.5–29.0)	24 (3–30)	0.226
ATS use, last 3 months (self-report)	55 (27.0)	12 (21.8)	0.569
Condom self-efficacy (median score; IQR)	21 (18–24)	20 (17–21)	0.035
Hormonal contraceptive use, last 3 months	22 (10.8)	1 (4.6)	0.088
Interpersonal factors			
Used condom at last sex (yes)	128 (62.8)	23 (18.0)	0.020
Condom use, last client (yes)	117 (90.0)	23 (19.7)	0.772
Condom use, last non-paying partner	11 (14.9)	0 (0.0)	0.393
Number of sexual partners, last month *Median, IQR*	4 (2–10)	3 (2–10)	0.790
Any client physical or sexual violence, last year	53 (26.0)	14 (26.4)	0.189
Any intimate partner physical or sexual violence, last year	41 (20.1)	9 (22.0)	0.957
Sex work factors			
Entertainment venue-based sex work, last month	131 (67.2)	24 (18.3)	0.241
Non-entertainment venue-based sex work, last month	64 (32.8)	16 (25.0)	–
Had a manager	145 (71.4)	29 (20.0)	0.172

**Table 2:** Pregnancy and HIV/STI prevention methods in the last 3 months, reported at baseline

	Total (100%) (*n* = 204)	Participants who were pregnant at least once over the study period	
Method for pregnancy prevention, last 3 months		Yes (20.1%) (*n* = 41)	No (79.9%) (*n* = 163)
Male condom	203 (99.5)	41 (20.2)	162 (79.8)
Withdrawal	71 (34.8)	11 (15.5)	60 (84.5)
Oral sex/sex without penetration	37 (18.1)	6 (16.2)	31 (83.8)
Birth control pills	16 (7.84)	1 (6.2)	15 (93.8)
Injectable contraceptives	6 (2.9)	0 (0.0)	6 (100.0)
Abortion	23 (11.3)	6 (26.1)	17 (73.9)
Other method	2 (0.98)	0 (0.0)	2 (100.0)
Method for HIV/STI prevention, last 3 months			
Male condom	204 (100.0)	41 (20.1)	163 (79.9)
Oral sex/sex without penetration	40 (19.6)	8 (20.0)	32 (80.0)
Masturbation	75 (36.8)	18 (24.0)	57 (76.0)

In multivariable analysis (Model 1), younger age (Adjusted Hazards Ratio (AHR): 2.28; 95% CI 1.22–4.27), and lower income (AHR 2.63; 95% CI 1.02–6.77) were independently associated with decreased time to pregnancy. Self-reported condom self-efficacy was associated with a longer time to incident pregnancy (AHR 0.89; 95% CI 0.81–0.98). As displayed in Table 3, similar associations were present in Model 2, a more parsimonious model restricted to factors significant at *p* < 0.05 in bivariate analysis.

**Table 3:** Multivariable Extended Cox analysis of correlates of incident pregnancy among 204 Female and Entertainment Sex workers (799 observations), working in Phnom Penh, Cambodia enrolled in the YWHS-II study

Characteristic	Unadjusted Hazard Ratio (HR)	Adjusted Hazard Ratio (AHR) MODEL 1 (FULL)	Adjusted Hazard Ratio (AHR) MODEL 2 (sig *p* < 0.05)
Socio-demographics, biological individual factors			
Age (years)			
16–18	1.00 (0.02–4.31)	1.59 (0.36–7.11)	1.37 (0.31–6.00)
19–24	2.28 (1.22–4.27)*	3.30 (1.57–6.96)^†^	2.56 (1.35–4.87)^†^
25–29 years	REF	REF	REF
Income, last month ($USD)			
≤ 150	2.29 (0.96–5.44)*	2.63 (1.02–6.77)^†^	–
> 150	REF	REF	–
Education			
No schooling	1.38 (0.40–4.70)	1.39 (0.38–5.12)	–
1–6 years	2.43 (0.86–6.89)*	1.87 (0.64–5.50)	–
7+ years	REF	REF	–
Marital status			
Legally married	2.09 (0.49–8.99)	1.42 (0.28–7.07)	1.76 (0.40–7.66)
Living together, not legally married	2.05 (1.09–3.84)^†^	1.92 (0.23–15.9)	1.95 (1.03–4.71)^†^
Single, widowed, divorced or separated	REF	REF	REF
Number of previous children	0.76 (0.56–1.03) ^†^	0.67 (0.38–1.17)	–
HIV positive	0.36 (0.11–1.16) ^†^	0.47 (0.13–1.63)	–
Days drank alcohol, last month	0.77 (0.51–1.18)	–	–
ATS use, last 3 months (self-report)	0.79 (0.35–1.78)	–	–
Condom self-efficacy‡	0.92 (0.85–0.99)^†^	0.89 (0.81–0.98)^†^	0.92 (0.84–0.99)^†^
Hormonal contraceptive use, last 3 months‡	5.63 (0.78–41.0)*	0.17 (0.02–1.27)	–
Interpersonal factors			
Condom use, last sex (yes)	0.45(0.23–0.88)^†^	0.57 (0.28–1.13)	–
Condom use, last client (yes)	0.74 (0.09–5.81)	–	–
Condom use, non-paying partner (yes)	0.53 (0.13–2.26)	–	–
Number of sexual partners, last month	1.11 (0.51–2.41)	–	–
Client physical or sexual violence, last year	1.88 (0.73–4.85)	–	–
Intimate partner physical or sexual violence, last year	0.96 (0.23–3.98)	–	–
Sex work factors			
Entertainment venue-based sex work, last month	0.67 (0.34–1.31)	–	–
Non- entertainment venue-based sex work, last month	REF	–	–
Had a manager	0.65 (0.35–1.21)	–	–

*p < 0.10

† *p* < 0.05

‡ at baseline

All variables (except condom self-efficacy) are time-updated to refer to occurrences in the past three months

## Discussion

The one-year pregnancy incidence in this cohort of young FESW was high, even when compared to Cambodia’s crude birth rate of 22 live births per 1000 women/year [23]. Moreover, despite the young age of our sample, the median number of children (three) in our cohort exceeded the estimated number of births for urban Cambodian women (2.1 children) over their entire reproductive lives [23]. The finding of high number of pregnancies corroborates a growing body of research, including unintended pregnancies (using abortion rates as a proxy), among women engaged in transactional sex in various settings [4, 8]. For example, 408/475 (86%) of women in transactional sex in Kenya and 264/514 (53%) of Colombian women engaged in sex work reported at least one induced abortion [2, 3]. In Uzbekistan, 109/448(24.3%) of women engaged in transactional sex reported three or more lifetime abortions [7]. While there is a need to examine pregnancy intentions among FESW in Cambodia, the high number of abortions in the past three months suggests that many pregnancies in this sample were unintended. Alongside previous research that documented 166/592 (28.2%) of brothel-based sex workers in Cambodia (in 2007) attending STI care in Phnom Penh had an induced abortion in the last year [24], these findings highlight large reproductive health needs among Cambodian women engaged in commercial sex.

The low use of hormonal contraceptives further underscores the reproductive health gap among FESW in this setting. The baseline prevalence of hormonal contraceptive use in our sample was roughly 23/204 (11%) compared to 642/2069 (31%) among Cambodian women living in urban areas [25]. This is especially low when compared to married women in Phnom Penh, (616/1099) 56% of whom were documented to use hormonal contraceptives in 2010 [25]. Previous research among brothel-based Cambodian women engaged in transactional sex revealed low SRH knowledge, with very few having attended family planning services [26]. Future research into the barriers to SRH services access, including hormonal contraceptives and safe abortion services is warranted. While hormonal contraceptive use in this sample was low, most participants reported using condoms to prevent pregnancy and HIV/STIs. This is not surprising given the dual function of condoms in preventing HIV/STIs and pregnancy, and their relative availability and low cost compared to hormonal contraceptives. The preference for condoms may also reflect Cambodia’s 100% condom use policy for women engaged in transactional sex work, previously enforced widely in brothels; however there is evidence that nation’s 2008 anti-trafficking legislation has impeded condom access and use [27]. Condoms as the sole method of contraception can be problematic, given their reduced effectiveness in preventing pregnancy compared to hormonal methods. Dual contraceptive methods (hormonal contraceptives in addition to condom use) are indicated to prevent both HIV/STI and pregnancy in this population. There is an urgent need to explore innovative sex worker-specific service delivery models to improve Cambodian FESWs’ access to non-judgmental SRH services that promote dual contraceptive use and safe abortion services.

FESW who reported increased self-efficacy or control over condom use had significantly lower pregnancy incidence, likely due to more consistent condom use by sexual partners. While FESWs’ ability to negotiate safe sex is critical, growing evidence suggests this needs to be coupled with venue-based interventions that provide access to condoms and support the safe negotiation of condom use by clients [27, 28]. It is well documented that condom negotiation and use is often beyond the control of women engaged in transactional sex, often due to gender-based violence (and fear of violence), and coerced unprotected sex [29–32]. In this analysis, client physical/sexual violence was associated with elevated pregnancy incidence, though this was not statistically significant. Multi-pronged combined interventions (e.g., safer indoor work spaces, supportive management, FESW mobilization and empowerment) [33] that offer protection against violence within environments that support condom negotiation and use are needed. Cambodia’s anti-trafficking legislation and the subsequent displacement of women formerly working in brothels to diverse and sometimes clandestine venues continues to act as an impediment to condom access, venue-based HIV prevention interventions [34] and undermines condom negotiation and access to HIV prevention and SRH services [16, 35]. Indeed, qualitative research among Cambodian FESW has revealed increased vulnerability to sexual and physical violence among those operating out of guesthouses and hotels, in part due to the lack of protection mechanisms previously offered by peers and management in more formal settings [27]. Barriers to HIV care service delivery have also been reported by NGOs following the legislative shift, as FESW have become more difficult to identify and reach [27].

The association between lower income and pregnancy incidence accords with literature documenting greater inconsistent condom use among women engaged in transactional sex with lower socioeconomic status [36]. Possible explanations for this association include the prohibitive costs of hormonal contraceptives and/or barriers to accessing reproductive health services. Lower-income FESW may also be more willing to accept client requests for unprotected sex in an effort to make more money per transaction. Finally, while the association between younger age (19–24 versus 25–29 years) and increased pregnancy incidence in our study warrants further investigation, possible explanations for this association include reduced knowledge of SRH, limited access to SRH services and supplies (e.g., condoms) and lower fertility intentions among older, parous FESW. Given the link between income and pregnancy incidence, structural approaches, such as microfinance or income-generating opportunities warrant investigation [37, 38].

Our findings also have important implications for HIV prevention trials that target FESW. Specifically, high pregnancy incidence may impact recruitment, retention and completion of HIV prevention trials, particularly interventions that require longer-term evaluations such as microbicides and PrEP. Indeed, almost one in ten (7.3%) women in our study were ineligible to initiate or complete (5.4%) the three-dose HPV vaccine series offered as part of the YWHS, due to pregnancy within the past six months [39]. These risk-related dropouts potentially bias study samples, impacting generalizability and, in the long term, the efficacy of related future interventions. Given the high burden of unintended pregnancy and low use of hormonal contraceptives among FESW in Cambodia (and elsewhere), there is a need to explore the potential of offering hormonal contraceptives to FESW not desiring pregnancy as part of future HIV prevention interventions.

Our analysis has several limitations. Firstly, the small sample size (and small number of incident pregnancies) may have limited the power of our multivariable analysis and the precision of our estimates. However, the use of multiple responses per participant in this longitudinal analysis increased the number of observations, tempering this bias. While the observational nature of this study precludes temporal inference (compared to an experimental study), the longitudinal design serves to strengthen the validity of these findings. Secondly, many of the variables in this analysis (including our pregnancy outcome) were self-reported and may be subject to social desirability bias. Despite this potential limitation, which would bias our results towards the null, significant associations with our outcome remained. Thirdly, we were unable to assess whether the incident pregnancies measured were intended or unintended. Future investigations into the pregnancy intentions/fertility desires of Cambodian FESW are needed. Fourthly, condom self-efficacy and contraceptive usage were only measured at baseline, and thus we were not able to account for changes in these variables over the follow-up period. Fifth, since the aim of the study was to examine pregnancy among women in entertainment and sex work venues with high sexual risk patterns, we did not collect data on nor were able to separate female sex workers from entertainment workers with high sexual risk in this analysis. Finally, the clandestine nature of sex work made systematic or probabilistic sampling a challenge, thus limiting the study’s generalizability to all young FESW in Cambodia. Despite this drawback, the current sample was able to capture a wide range of FESW working in a variety of sex work settings across Phnom Penh.

## Conclusions

Our results highlight a high incidence of pregnancy and unmet reproductive health need among FESW, with younger age, lower income and low condom-self efficacy associated with reduced time to the occurrence of pregnancy. These findings suggest a need for combined interventions to increase access to SRH services. To be successful, such interventions would need to be supported by legislative and policy shifts that permit FESW to work in safer, more supportive formal indoor settings [29, 33]. Finally, the high level of pregnancy among FESW observed in the current study has important implications for the SRH rights of FESWs and the recruitment, retention and the success of HIV prevention trials targeting FESW. There is a need to offer SRH information and services to potential HIV prevention trials participants not desiring pregnancy. Access to SRH services may improve trial recruitment and retention and, more importantly, help support the unmet reproductive rights of FESWs.

## Abbreviations

ATS: Amphetamine-type stimulants
CWDA: Cambodian Women’s Development Association
FESW: Female and Entertainment Sex Workers
HIV: Human Immunodeficiency Virus
HIV/AIDS: Human Immunodeficiency Virus/ Acquired immunodeficiency syndrome
HPV: Human papillomavirus
NCHADS: the National Center for HIV/AIDS, Dermatology and STD
SRH: Sexual and Reproductive Health
STI: Sexually Transmitted Infections
UCSF: the University of California, San Francisco
UNSW: University of New South Wales
YWHS: Young Women’s Health Study
